# Financial Toxicity and Its Association with the Quality of Life of Greek Patients with Cancer: A Cross-Sectional Study

**DOI:** 10.3390/nursrep15020067

**Published:** 2025-02-13

**Authors:** Athanasios Pitis, Maria Diamantopoulou, Aspasia Panagiotou, Dimitrios Papageorgiou, Foteini Tzavella

**Affiliations:** Department of Nursing, School of Health Sciences, University of Peloponnese, 22131 Tripoli, Greece; m.diamantopoulou@office365.uop.gr (M.D.); aspasi@uop.gr (A.P.); dpapageorg@uop.gr (D.P.); tzavella@uop.gr (F.T.)

**Keywords:** financial toxicity, quality of life, cancer, nursing

## Abstract

**Background/Objectives**: Greek cancer patients deal with high out-of-pocket medical expenses in comparison with the European Union average. All these high costs affect the quality of life of cancer patients, leading to financial toxicity. The purpose of this study is to investigate the association between financial toxicity and quality of life in patients undergoing cancer treatment. **Methods**: A cross-sectional study was conducted in four hospitals in Greece. The Comprehensive Score for Financial Toxicity (COST) Scale was used for the evaluation of financial toxicity, and the EORTC Core Quality of Life questionnaire (EORTC QLQ-C30) was used for the assessment of quality of life. Quantitative variables were first tested for normality using the Kolmogorov–Smirnov criterion. Spearman correlation coefficients (rho) were used to explore the association of two continuous variables. Multiple linear regression analysis was used with dependent the QoL subscales. Logarithmic transformations of the QoL scales were used for the regression analyses. Internal consistency reliability was determined by the calculation of Cronbach’s α coefficient. All reported *p* values are two-tailed. Statistical significance was set at *p* < 0.05 and analyses were conducted using SPSS statistical software (version 27.0). **Results**: Greater financial toxicity score, i.e., lower toxicity, was significantly associated with greater global health status (rho = 0.34; *p* < 0.001) and greater physical (rho = 0.37; *p* < 0.001), role (rho = 0.17; *p* = 0.001), emotional (rho = 0.34; *p* < 0.001), cognitive (rho = 0.22; *p* < 0.001), and social (rho = 0.27; *p* < 0.001) functioning. **Conclusions**: There is a strong correlation between a greater financial toxicity score and the quality of life of Greek cancer patients, meaning the lower their financial toxicity, the better their quality of life.

## 1. Introduction

Following the beginning of the financial crisis in 2009, Greece, due to increased national debt, accepted an economic memorandum, which highly impacted the healthcare sector [1]. As of 2022, the number of new cancer cases in Greece was 65.703, and the number of people who died of cancer that year was 32,385 [2]. In 2018, the total financial burden of cancer in Greek patients was €188 per capita, most of which was due to healthcare expenses [3]. Greece has a high percentage of out-of-pocket medical expenses, representing 33% of healthcare costs in 2021, while the average cost in the European Union was 18%. Although healthcare services in Greece, including cancer care, are free [4], many patients who choose the national public health system are being devastated because of the high out-of-pocket costs. As a result, they experience economic burden, devastating their overall well-being.

All these additional medical expenses patients and their families cope with may culminate in financial toxicity [5], which, according to the National Cancer Institute, is “a term used to describe problems a patient has related to the cost of medical care”. Financial difficulties might result from a lack of health insurance or from high medical bills that are not compensated by insurance, which can give rise to debt and bankruptcy. Access to healthcare and a patient’s quality of life can both be negatively impacted by financial toxicity. To conserve money, a patient could, for instance, refuse to comply with drug prescriptions or postpone doctor appointments. Financial toxicity is more common in oncology patients than in non-cancer individuals. Other names include “economic burden, economic hardship, financial burden, financial distress, financial hardship, and financial stress” [6,7]. World Health Organization (WHO) defines quality of life (QoL) as “an individual’s perception of their position in life in the context of the culture and value systems in which they live and in relation to their goals, expectations, standards and concerns” [8], while “health-related quality of life” (HRQoL) focuses QoL on health-related aspects. Nevertheless, HRQoL is a broad and complicated term for which no commonly recognized definition exists [9]; available definitions vary between those prioritizing physical, social, and emotional well-being and those emphasizing the influence of an individual’s health on everyday living [10]. Unlike QoL, HRQoL may incorporate both subjective and objective viewpoints in each area [11].

Numerous studies have revealed multiple determinants of quality of life (QOL) among cancer patients. Financial toxicity, defined “as the subjective and objective financial burden and suffering of cancer patients due to novel medical therapies and associated health services” [12], appears to be a new factor connected to QOL of oncology populations, and research indicates an unfavorable association of financial toxicity and quality of life indicators, such as both scores regarding mental and physical health [13,14,15].

Due to the high out-of-pocket healthcare costs cancer patients face in Greece, we hypothesized that there would be a high prevalence of financial toxicity in Greek cancer patients and that HRQoL would be impacted and be lower in those patients. Thus, this study aims to evaluate the association between financial toxicity and quality of life in patients undergoing cancer treatment.

## 2. Materials and Methods

### 2.1. Participants

Participants in the study were recruited while receiving treatment from the Oncology Day Clinics of the Theageneio Anticancer Hospital of Thessaloniki, General Oncology Hospital of Kifisia Agioi Anargiroi, Athens General Hospital of Thoracic Diseases Sotiria, and General Hospital of Athens Ippokrateio between April 2023 and April 2024. Convenience sampling was used to recruit participants. In order to be recruited, participants should be at least 18 years of age, speak and write Greek fluently, be patients with solid tumors, be receiving treatment at the time of the study for at least 4 weeks, and be able to give informed consent. The exclusion criteria included patients with hematological malignancies and patients with cognitive and behavioral difficulties that could limit their capability of completing the evaluation instruments. A total of 538 questionnaires were distributed, and 400 were returned.

### 2.2. Measurements

#### 2.2.1. Sociodemographic and Clinical Characteristics

A questionnaire regarding sociodemographic and clinical characteristics such as gender, educational level, family status, economic status, type of cancer, metastasis, cancer stage, and type(s) of treatment was used. Type of cancer, metastatic disease, and type(s) of treatment were confirmed by assessing the patients’ charts. Regarding the economic status, a 5-point Likert Scale was used.

#### 2.2.2. Financial Toxicity

For the assessment of financial toxicity, the validated Greek version of COST: A FACIT Measure of Financial Toxicity (FACIT-COST) was used, which is a patient self-completed tool that is used to evaluate the financial toxicity experienced by oncology patients, on a 5-point scale with 0 meaning “not at all”, 1 meaning “a little bit”, 2 meaning “somewhat”, 3 meaning “quite a bit”, and 4 meaning “very much”. The financial toxicity score was computed by adding up the 11 items, multiplying by 11, and dividing by the number of items answered. Items 2, 3, 4, 5, 8, 9, and 10 were reverse-scored. The higher the score, the better the financial well-being. The Comprehensive Score for Financial Toxicity (COST) Scale is a reliable tool, both in its original [16] and the validated Greek version [17].

#### 2.2.3. Quality of Life

For the assessment of quality of life, the validated Greek-language EORTC Core Quality of Life questionnaire (EORTC QLQ-C30) was used. It is self-administered, and the questions refer to the patient’s state during the last week. It consists of 30 items, which measure five functional scales (physical, social, emotional, cognitive, and role functioning), three symptom scales (fatigue, pain, nausea, and vomit), six single-item symptom scales assessing other cancer-related symptoms (dyspnoea, insomnia, appetite loss, constipation, diarrhea, and economic impact) and its treatment, and a global health status/QoL scale. The first 28 items present Likert-type responses, with a four-point format: “not at all”, “a little”, “quite a bit”, and “very much”. Items 29 and 30 are global scales with seven possible responses, ranging from 1—“very poor” to 7—“excellent”. Higher values on the symptom scale indicate a decrease in QoL due to cancer-associated symptoms, while higher values on the global and functional health scales represent better QoL. The questionnaire was well accepted in the Greek population and had very high reliability [18].

### 2.3. Statistical Analysis

Quantitative variables were first tested for normality using the Kolmogorov–Smirnov criterion. Quantitative variables were expressed as mean values (standard deviation) and as median (interquartile range), while categorical variables were expressed as absolute and relative frequencies. Spearman correlation coefficients (rho) were used to explore the association of two continuous variables. The coefficient is considered very high when it is above 0.9, high when it is 0.7–0.9, moderate when it is 0.5–0.7, low when it is 0.3–0.5, and very low when it is below 0.3 [19]. Multiple linear regression analysis was used with dependent the QoL subscales. The regression equation included terms for patients’ demographical and clinical characteristics, as well as their financial toxicity score. Adjusted regression coefficients (β) with standard errors (SE) were computed from the results of the linear regression analyses. Logarithmic transformations of the QoL scales were used for the regression analyses. Internal consistency reliability was determined by the calculation of Cronbach’s α coefficient. Scales with reliabilities equal to or greater than 0.70 were considered acceptable. All reported *p*-values are two-tailed. Statistical significance was set at *p* < 0.05, and analyses were conducted using SPSS statistical software (version 27.0).

## 3. Results

### 3.1. Sociodemographic and Clinical Characteristics

Data from 400 patients were collected and analyzed. The survey response rate was 74.3%. Their demographic and clinical characteristics are presented in Table 1. The mean age was 61.7 years (SD = 12.5 years). Almost half of the patients (50.8%) were female, 60.0% were married, and 79.3% had children; 51.1% of the sample were moderately satisfied with their economic status; 45.5% of patients had metastatic cancer, 45.3% were at stage IV, and almost all had undergone chemotherapy (99.8%).

### 3.2. Descriptive Measures and Reliability Indexes

Descriptive measures and reliability indexes of QoL and financial toxicity scales are presented in Table 2. The mean global health status score was 59.35% (SD = 22.02%), and the mean financial toxicity score was 20.83 (SD = 7.90). All reliability indexes were above 0.7, indicating acceptable reliability.

### 3.3. Correlational Analysis

Greater financial toxicity score, i.e., lower toxicity, was significantly associated with greater global health status (rho = 0.34; *p* < 0.001), greater physical (rho = 0.37; *p* < 0.001), role (rho = 0.17; *p* = 0.001), emotional (rho = 0.34; *p* < 0.001), cognitive (rho = 0.22; *p* < 0.001), and social (rho = 0.27; *p* < 0.001) functioning (Table 3). Greater financial toxicity score was significantly associated with less fatigue (rho = −0.30; *p* < 0.001), nausea and vomiting (rho = −0.13; *p* = 0.010), pain (rho = −0.27; *p* < 0.001), dyspnea (rho = −0.28; *p* < 0.001), insomnia (rho = −0.15; *p* = 0.004), diarrhea (rho = −0.10; *p* = 0.049) symptoms and less financial difficulties (rho = −0.57; *p* < 0.001).

The aforementioned results remained significant after adjusting for demographical and clinical parameters, as shown in Table 4 and Table 5. Moreover, as far as the rest of the characteristics are concerned, it was found that among all symptom subscales (Table 4), only gender was significantly associated with nausea and vomiting symptoms (β = 0.131; *p* = 0.045), with women having more symptoms. Also, greater age was significantly associated with worse global health status (β = −0.003; *p* = 0.011) and worse physical functioning (β = −0.002; *p* = 0.001). Patients with children had significantly better global health status (β = 0.083; *p* = 0.020), and females showed significantly worse physical functioning compared to men (β = −0.039; *p* = 0.019).

## 4. Discussion

The current study’s findings indicate a significant association between greater financial toxicity score, i.e., lower toxicity, and greater global health status (rho = 0.34; *p* < 0.001) role (rho = 0.17; *p* = 0.001), emotional (rho = 0.34; *p* < 0.001), cognitive (rho = 0.22; *p* < 0.001), and social (rho = 0.27; *p* < 0.001) functioning (Table 3). Meanwhile, greater financial toxicity score was significantly associated with less fatigue (rho = −0.30; *p* < 0.001), nausea and vomiting (rho = −0.13; *p* = 0.010), pain (rho = −0.27; *p* < 0.001), dyspnea (rho = −0.28; *p* < 0.001), insomnia (rho = −0.15; *p* = 0.004), and diarrhea (rho = −0.10; *p* = 0.049) symptoms and less financial difficulties (rho = −0.57; *p* < 0.001).

These results are in line with the results of a recent systematic review and meta-analysis that revealed HRQoL was significantly greater in oncology patients exhibiting lesser financial toxicity [14]. Additionally, recent studies signify a strong correlation between better quality of life and lower financial toxicity, i.e., greater financial toxicity scores [20]. A cross-sectional study in Greek patients with lung cancer found a strong association between HRQoL and financial welfare [17].

Similar results were observed in studies carried out in China, where participants suffered from financial distress, which impacted their HRQoL [21], and in North Carolina, where cancer patients coping with financial hardship experienced lower quality of life [22]. As for patients experiencing new financial toxicity, their health-related quality of life seems to worsen [23]. Equivalent results were found in a study conducted in India on oncology patients receiving palliative care; it was discovered there was a strong association between financial toxicity and HRQoL with most of the patients being married and requiring economic assistance [24].

Additionally, the results of this study are in line with those of a study carried out in Brazil during the COVID-19 pandemic. A significant association was figured out between HRQoL and financial toxicity, with results indicating domains of emotional, functional, and physical well-being being significant [25]. On the contrary, it is interesting that two studies that were conducted in Japan suggested that there was a weak correlation between greater financial toxicity scores and overall health-related quality of life/global health status scores, whereas it was significantly correlated with lower health-related quality of life scores in particular features [13,15]. However, a significant association between greater financial score and certain aspects of quality of life, such as greater physical, role, emotional, cognitive, and social functioning [15], and financial difficulties [13,15], was found, results that are in accordance with those of previous research and this one. This could be easily explained on the grounds that the Japanese healthcare system differs significantly in comparison to the Greek healthcare system. Since the financial crisis, governments have enhanced control and made access to medical care more strict in an attempt to reduce medical, pharmaceutical, and overall healthcare expenses [1]. At the same time, the recent COVID-19 pandemic and the ongoing war in Eastern Europe further increased poverty and economic and social disparities [26]. Regarding healthcare professionals’ point of view in Greece, the several cutbacks in their income, the increasing flow of patients in the emergency room, and the low nurse-to-patient frequency in the public national health system have led to a vicious cycle of inadequate working circumstances and poorer quality of medical services [1].

Our results revealed that the milder the physical symptoms, the lower the financial toxicity patients with cancer are dealing with. The results of other studies also support these findings [27,28,29]. Women seem to be more affected by having more symptoms of nausea and vomiting in the current study; recent studies coincide with these results, suggesting that men withstand better physical symptoms like nausea and vomiting [24,27,28].

These observations underline the necessity for customized interventions and assistance for patients coping with cancer in order to manage financial toxicity and improve the quality of life of cancer patients. More importantly, health professionals, especially nurses who share more time with patients during cancer care, should use quick instruments that assess the economic hardship that Greek cancer patients may face and refer them for financial counseling. Furthermore, it is important to address specific domains of QoL that are impacted by financial toxicity, such as social, cognitive, emotional, and role functioning, and develop interventions regarding symptom management in cancer patients.

The cross-sectional design was one of the study’s major limitations; secondly, it would be beneficial for future research studies to address financial toxicity at the beginning of the treatment and after months of receiving treatment to better recognize its impact on quality of life; moreover, financial toxicity could be evaluated by specific cancer type or treatment in future research. Future studies may also investigate the development of nurse-led interventions regarding counseling and management of financial toxicity in cancer patients for better quality-of-life outcomes.

## Figures and Tables

**Table 1:** Sample’s characteristics.

n = 400		n (%)
Demographical characteristics	Gender	
	Male	197 (49.3)
	Female	203 (50.8)
	Age, mean (SD)	61.7 (12.5)
	Educational level	
	Primary school	71 (17.8)
	Middle school	46 (11.5)
	High school	122 (30.5)
	Technical university	46 (11.5)
	University	73 (18.3)
	MSc	33 (8.3)
	PhD	9 (2.3)
	Family status	
	Unmarried	54 (13.5)
	Married	240 (60.0)
	Civil partnership	6 (1.5)
	Divorced	48 (12.0)
	Separated	6 (1.5)
	Widowed	46 (11.5)
	Children	317 (79.3)
	Satisfaction from economic status	
	Not at all	62 (15.7)
	A little	52 (13.2)
	Moderately	202 (51.1)
	Much	67 (17.0)
	Very much	12 (3.0)
Clinical characteristics	Type of cancer	
	Gastrointestinal cancers	92 (23.0)
	Head and neck cancers	7 (1.8)
	Sarcomas	19 (4.8)
	Gynecological cancers	22 (5.5)
	Lung cancer	110 (27.5)
	Breast cancer	89 (22.3)
	Bladder cancer	20 (5.0)
	Pancreatic cancer	29 (7.3)
	Other	12 (3.0)
	Metastatic	182 (45.5)
	Disease stage	
	Ι	30 (7.5)
	ΙΙ	95 (23.8)
	ΙΙΙ	94 (23.5)
	IV	181 (45.3)
	Surgery	240 (60)
	Chemotherapy	399 (99.8)
	Radiotherapy	92 (23.0)
	Immunotherapy	139 (34.8)

**Table 2:** Descriptive measures and reliability indexes of QoL and financial toxicity scales.

	Minimum	Maximum	Mean (SD)	Median (IQR)	Cronbach’s Alpha
Global health status/QoL ^1^	0.00	100.00	59.35 (22.02)	58.33 (50.00–75.00)	0.80
Fatigue ^2^	0.00	100.00	46.69 (29.37)	44.44 (22.22–66.67)	0.83
Nausea and vomiting ^2^	0.00	100.00	9.63 (19.63)	0.00 (0.00–16.67)	0.70
Pain ^2^	0.00	100.00	26.82 (30.91)	16.67 (0.00–50.00)	0.87
Dyspnoea ^2^	0.00	100.00	34.83 (33.17)	33.33 (0.00–66.67)	-
Insomnia ^2^	0.00	100.00	36.25 (36.86)	33.33 (0.00–66.67)	-
Appetite loss ^2^	0.00	100.00	19.75 (31.33)	0.00 (0.00–33.33)	-
Constipation ^2^	0.00	100.00	21.00 (32.07)	0.00 (0.00–33.33)	-
Diarrhea ^2^	0.00	100.00	17.75 (29.83)	0.00 (0.00–33.33)	-
Financial difficulties ^2^	0.00	100.00	35.58 (34.25)	33.33 (0.00–66.67)	-
Physical functioning ^1^	0.00	100.00	66.93 (22.31)	66.67 (53.33–86.67)	0.80
Role functioning ^1^	0.00	100.00	59.21 (32.50)	66.67 (33.33–83.33)	0.81
Emotional functioning ^1^	0.00	100.00	69.48 (24.35)	75.00 (58.33–91.67)	0.71
Cognitive functioning ^1^	0.00	100.00	81.42 (23.93)	83.33 (66.67–100.00)	0.74
Social functioning ^1^	0.00	100.00	61.38 (32.13)	66.67 (33.33–91.67)	0.79
Financial Toxicity Score (COST) ^3^	4.00	40.00	20.83 (7.90)	20.00 (15.00–27.00)	0.78

Note. In scales with one item, Cronbach’s alpha coefficient could not be computed. ^1^ Greater values indicate better QoL; ^2^ greater values indicate worse QoL; ^3^ greater values indicate less toxicity.

**Table 3:** Spearman’s correlation coefficients (rho) between QoL and financial toxicity scales.

	Financial Toxicity Score (COST)	
	rho	*p*
Global health status/QoL	0.34	<0.001
Fatigue	−0.30	<0.001
Nausea and vomiting	−0.13	0.010
Pain	−0.27	<0.001
Dyspnoea	−0.28	<0.001
Insomnia	−0.15	0.004
Appetite loss	−0.09	0.061
Constipation	−0.04	0.385
Diarrhea	−0.10	0.049
Financial difficulties	−0.57	<0.001
Physical functioning	0.37	<0.001
Role functioning	0.17	0.001
Emotional functioning	0.34	<0.001
Cognitive functioning	0.22	<0.001
Social functioning	0.27	<0.001

**Table 4:** Multiple linear regression analysis results with symptoms subscales as dependent variables.

	Fatigue			Nausea and Vomiting			Pain		Dyspnoea	
	β (SE)+		*p*	β (SE)+	*p*		β (SE)+	*p*	β (SE)+	*p*
Gender (Female vs. Male)	0.099 (0.058)		0.088	0.131 (0.051)	0.045		0.063 (0.085)	0.464	−0.089 (0.087)	0.308
Age	0.001 (0.003)		0.599	−0.004 (0.003)	0.155		−0.004 (0.004)	0.316	−0.005 (0.004)	0.171
Educational level ^1^	0.038 (0.019)		0.054	0.025 (0.024)	0.290		0.016 (0.029)	0.568	−0.013 (0.029)	0.647
Married/Civil partnership (Yes vs. No)	−0.021 (0.062)		0.733	−0.060 (0.076)	0.428		0.028 (0.091)	0.754	−0.079 (0.093)	0.400
Children (Yes vs. No)	0.047 (0.076)		0.539	0.038 (0.093)	0.681		0.106 (0.112)	0.345	0.001 (0.115)	0.994
Satisfaction from economic status ^2^	0.068 (0.036)		0.058	0.041 (0.044)	0.353		0.077 (0.053)	0.143	0.079 (0.054)	0.144
Metastatic (Yes vs. No)	0.011 (0.100)		0.909	−0.019 (0.122)	0.874		0.011 (0.146)	0.938	0.155 (0.150)	0.302
Disease stage ^3^	0.070 (0.050)		0.167	0.105 (0.062)	0.088		0.097 (0.074)	0.191	−0.003 (0.076)	0.969
Surgery (Yes vs. No)	0.032 (0.057)		0.568	−0.048 (0.069)	0.489		−0.091 (0.083)	0.274	−0.019 (0.085)	0.819
Radiotherapy (Yes vs. No)	−0.011 (0.066)		0.867	−0.021 (0.081)	0.797		0.090 (0.097)	0.356	−0.065 (0.100)	0.516
Immunotherapy (Yes vs. No)	0.052 (0.059)		0.376	−0.050 (0.072)	0.482		0.021 (0.086)	0.810	0.073 (0.088)	0.411
Financial Toxicity Score (COST)	−0.025 (0.005)		<0.001	−0.015 (0.006)	0.007		−0.034 (0.007)	<0.001	−0.031 (0.007)	<0.001
	**Insomnia**		**Appetite loss**		**Constipation**		**Diarrhea**		**Financial** **difficulties**	
	**β (SE)+**	***p***	**β (SE)+**	***p***	**β (SE)+**	***p***	**β (SE)+**	***p***	**β (SE)+**	***p***
Gender (Female vs. Male)	0.138 (0.093)	0.139	−0.043 (0.088)	0.625	0.136 (0.091)	0.134	0.062 (0.086)	0.468	0.012 (0.078)	0.876
Age	−0.001 (0.004)	0.867	0.004 (0.004)	0.258	0.002 (0.004)	0.624	0.002 (0.004)	0.550	−0.006 (0.003)	0.090
Educational level ^1^	0.034 (0.031)	0.282	0.033 (0.030)	0.268	−0.007 (0.030)	0.813	0.061 (0.039)	0.074	−0.011 (0.026)	0.684
Married/Civil partnership (Yes vs. No)	−0.114 (0.100)	0.254	0.071 (0.094)	0.450	0.070 (0.097)	0.466	−0.087 (0.092)	0.342	−0.114 (0.083)	0.171
Children (Yes vs. No)	0.080 (0.122)	0.515	−0.063 (0.115)	0.586	−0.168 (0.119)	0.159	−0.037 (0.112)	0.741	−0.039 (0.102)	0.706
Satisfaction from economic status ^2^	−0.003 (0.058)	0.953	0.098 (0.054)	0.072	−0.057 (0.056)	0.310	−0.011 (0.053)	0.838	0.009 (0.048)	0.847
Metastatic (Yes vs. No)	−0.067 (0.160)	0.676	−0.079 (0.151)	0.600	−0.094 (0.155)	0.544	0.112 (0.147)	0.449	0.092 (0.134)	0.493
Disease stage ^3^	0.071 (0.081)	0.384	0.108 (0.076)	0.159	0.030 (0.078)	0.706	0.007 (0.074)	0.920	0.009 (0.068)	0.899
Surgery (Yes vs. No)	0.006 (0.091)	0.951	−0.043 (0.086)	0.617	0.030 (0.088)	0.735	0.010 (0.084)	0.905	0.070 (0.076)	0.360
Radiotherapy (Yes vs. No)	−0.046 (0.106)	0.665	0.027 (0.101)	0.790	0.150 (0.103)	0.146	0.037 (0.098)	0.708	−0.047 (0.089)	0.597
Immunotherapy (Yes vs. No)	0.040 (0.094)	0.674	0.118 (0.089)	0.184	0.046 (0.091)	0.613	−0.159 (0.087)	0.068	0.034 (0.079)	0.668
Financial Toxicity Score (COST)	−0.016 (0.007)	0.030	−0.010 (0.005)	0.056	−0.003 (0.007)	0.695	−0.013 (0.007)	0.050	−0.054 (0.006)	<0.001

Note. Analyses were made after logarithmically transforming the dependent variables. + Regression coefficient (Standard Error). ^1^ Higher values indicate greater educational level; ^2^ higher values indicate greater satisfaction; ^3^ higher values indicate greater stage.

**Table 5:** Multiple linear regression analysis results with global health status and functioning subscales as dependent variables.

	Global Health Status/QoL		Physical Functioning		Role Functioning		Emotional Functioning		Cognitive Functioning		Social Functioning	
	β (SE)+	*p*	β (SE)+	*p*	β (SE)+	*p*	β (SE)+	*p*	β (SE)+	*p*	β (SE)+	*p*
Gender (Female vs. Male)	−0.038 (0.027)	0.168	−0.039 (0.019)	0.043	−0.006 (0.059)	0.922	−0.032 (0.031)	0.298	−0.054 (0.030)	0.070	−0.061 (0.059)	0.306
Age	−0.003 (0.001)	0.011	−0.002 (0.001)	0.021	−0.002 (0.003)	0.548	0.000 (0.001)	0.983	0.000 (0.001)	0.759	−0.003 (0.003)	0.183
Educational level ^1^	0.004 (0.009)	0.647	0.001 (0.006)	0.921	−0.030 (0.020)	0.123	0.010 (0.010)	0.325	0.012 (0.010)	0.227	−0.025 (0.020)	0.207
Married/Civil partnership (Yes vs. No)	0.014 (0.029)	0.638	0.016 (0.021)	0.448	0.062 (0.063)	0.325	0.040 (0.033)	0.218	−0.013 (0.032)	0.693	0.118 (0.063)	0.062
Children (Yes vs. No)	0.083 (0.036)	0.020	0.022 (0.025)	0.380	0.131 (0.077)	0.090	0.077 (0.040)	0.054	−0.023 (0.039)	0.561	−0.053 (0.078)	0.492
Satisfaction from economic status ^2^	0.003 (0.017)	0.856	0.008 (0.012)	0.487	−0.047 (0.036)	0.201	0.013 (0.019)	0.493	−0.002 (0.018)	0.926	−0.032 (0.037)	0.386
Metastatic (Yes vs. No)	−0.023 (0.046)	0.615	−0.049 (0.033)	0.140	−0.065 (0.101)	0.520	−0.036 (0.052)	0.490	−0.011 (0.051)	0.824	−0.118 (0.101)	0.247
Disease stage ^3^	−0.035 (0.024)	0.134	−0.018 (0.017)	0.284	−0.030 (0.051)	0.555	−0.029 (0.026)	0.269	−0.028 (0.026)	0.273	−0.044 (0.051)	0.395
Surgery (Yes vs. No)	0.016 (0.026)	0.536	0.023 (0.019)	0.219	0.104 (0.057)	0.071	0.030 (0.030)	0.321	−0.025 (0.029)	0.395	0.025 (0.058)	0.660
Radiotherapy (Yes vs. No)	0.011 (0.031)	0.713	−0.030 (0.022)	0.168	−0.055 (0.067)	0.417	−0.042 (0.035)	0.232	0.001 (0.034)	0.975	−0.071 (0.068)	0.294
Immunotherapy (Yes vs. No)	0.009 (0.027)	0.733	0.011 (0.019)	0.570	0.023 (0.059)	0.704	0.021 (0.031)	0.494	−0.041 (0.030)	0.169	0.090 (0.060)	0.131
Financial Toxicity Score (COST)	0.008 (0.002)	<0.001	0.006 (0.002)	<0.001	0.016 (0.005)	<0.001	0.009 (0.002)	<0.001	0.005 (0.002)	0.027	0.017 (0.005)	<0.001

Note. Analyses were made after logarithmically transforming the dependent variables. ^1^ Higher values indicate greater educational level; ^2^ higher values indicate greater satisfaction; ^3^ higher values indicate greater stage.

## Data Availability

All data provided and analyzed during this study are available in this paper. Additional data are available from the corresponding author upon reasonable request.

## Public Involvement Statement

There was no public involvement in any aspect of this research.

## Guidelines and Standards Statement

This manuscript was drafted following the STROBE guidelines for cross-sectional studies.
